# Editorial: Transgenerational effects of endocrine disrupting chemicals: unraveling hormonal and health impacts across generations

**DOI:** 10.3389/fendo.2026.1878580

**Published:** 2026-05-21

**Authors:** Jones B. Graceli, Min Nian

**Affiliations:** 1 Departamento de Morfologia, Universidade Federal do Espírito Santo, Vitória, ES, Brazil; 2 Animal Science, School of Agricultural Sciences, Southern Illinois University, Carbondale, IL, United States; 3 Department of Environmental Health, School of Public Health, Shanghai Jiao Tong, University School of Medicine, Shanghai, China

**Keywords:** critical windows of susceptibility, developmental toxicity, endocrine-disrupting chemicals (EDCs), epigenetic modifications, transgenerational effects

Endocrine-disrupting chemicals (EDCs) remain a major concern in environmental endocrinology because they can interfere with hormone synthesis, metabolism, transport, receptor signaling, and downstream developmental processes. Current evidence indicates that EDC exposure may affect not only directly exposed individuals but also subsequent generations, particularly when exposure occurs during sensitive developmental windows (1). The present Research Topic, *“Transgenerational Effects of Endocrine Disrupting Chemicals: Unraveling Hormonal and Health Impacts Across Generations,”* brings together experimental, epidemiological, and review evidence addressing how EDCs may influence endocrine, metabolic, neurodevelopmental, and reproductive outcomes across generations.

Over the past decades, EDC research has shown that these chemicals can interfere with hormone synthesis, secretion, transport, metabolism, and receptor-mediated signaling, with effects across multiple organ systems (1). More recent work has moved the field beyond direct toxicity and toward developmental programming, especially when exposure occurs during embryonic, fetal, or early postnatal life (2, 3). These periods are particularly important because endocrine signals help guide organ development, brain sexual differentiation, metabolic regulation, and reproductive maturation. Disruption during these windows may therefore have consequences that persist into later life and, in some cases, may extend to subsequent generations through epigenetic or germline-related mechanisms (4).

Several articles in this Research Topic focus on neuroendocrine development and behavior. The review by Dias et al. discussed heavy metals, including methylmercury, lead, and cadmium, as environmental toxicants with endocrine-disrupting properties. These metals may affect neurogenesis, synaptic plasticity, thyroid and steroid hormone signaling, and oxidative stress pathways, thereby contributing to impaired learning and memory. Evidence from prenatal exposure studies further suggests that early-life metal exposure may influence cognitive outcomes in later generations. This work broadens the scope of EDC research by showing that neuroendocrine disruption is not limited to classical organic pollutants.

The review by Glaecir et al. further examined classical EDCs, including bisphenols, phthalates, and vinclozolin, in relation to brain development and behavior. The authors summarized evidence that exposure during pre-, peri-, and postnatal periods can alter sexual differentiation of the brain, endocrine signaling, and behavioral outcomes. These findings are relevant because these compounds are widely used in consumer and industrial products, leading to frequent human exposure. Together, these two neurodevelopment-focused reviews indicate that the developing brain is a sensitive target of endocrine disruption and that both chemical class and timing of exposure may shape later neurobehavioral risk.

Metabolic health is another major theme of this Research Topic. Yu et al. used transcriptomic and metabolomic approaches to examine the effects of early-life perfluorooctanesulfonic acid (PFOS) exposure in adult offspring. Their study reported impaired glucose tolerance, altered lipid metabolism, and changes in insulin and peroxisome proliferator-activated receptor (PPAR) signaling pathways. The combined omics results suggest that developmental PFOS exposure may leave persistent molecular signatures related to metabolic homeostasis. These findings support the view that metabolic disorders may have environmental and developmental origins, especially when exposure occurs during periods of rapid growth and endocrine maturation.

The systematic review by Mitra et al. adds epidemiological evidence by examining phthalate exposure and gestational diabetes mellitus. The review highlights urinary phthalate metabolites as exposure biomarkers associated with gestational diabetes mellitus (GDM) risk. Because GDM affects both maternal health and offspring metabolic development, this evidence links pregnancy exposure to a broader intergenerational metabolic pathway. When considered together with the PFOS study, these findings suggest that EDCs may affect metabolic health through both direct developmental programming and pregnancy-related complications. Reproductive health, particularly male fertility, is also addressed in this Research Topic. Basak et al. discuss the potential transgenerational effects of bisphenol A (BPA) and its analogs on male infertility, with attention to gut dysbiosis, epigenetic regulation, and endocrine disruption. BPA-related changes in the gut microbiome may affect inflammation, hormone metabolism, and systemic metabolic status, all of which can influence reproductive function. Epigenetic changes induced during sensitive developmental windows may provide another route through which reproductive effects persist across generations. This perspective highlights the need to study male reproductive toxicity not only through the hypothalamic-pituitary-gonadal axis, but also through gut-derived, immune, and epigenetic pathways.

These articles also point to a broader issue in EDC research. Humans are rarely exposed to a single chemical at a time. Mixture exposure may produce additive, synergistic, or antagonistic effects, making it difficult to predict risk from single-chemical data alone (5). This challenge is particularly relevant for EDCs because low-dose effects, non-monotonic dose-response relationships, and developmental timing can complicate traditional risk assessment (6). Recent discussions on EDC regulation have therefore emphasized the need to incorporate mixture effects, developmental susceptibility, and long-term outcomes into public health decision-making (7).

Despite progress, several gaps remain (4). First, intergenerational and true transgenerational effects need to be distinguished more clearly. In pregnancy exposure models, the mother, fetus, and fetal germ cells may all be directly exposed, which complicates interpretation of effects observed in later generations. Second, most evidence for transgenerational inheritance still comes from animal studies, while human studies with multi-generation follow-up remain limited (8, 9). Third, the molecular mechanisms linking EDC exposure to persistent health effects require further validation. DNA methylation, histone modifications, non-coding RNAs, transcriptomic changes, metabolomic profiles, and microbiome alterations are all plausible mechanisms, but their stability and causal roles across generations are not yet fully established (9)

In conclusion, this Research Topic highlights how EDC exposure during sensitive developmental windows may affect neurodevelopment, metabolism, pregnancy outcomes, and reproductive health. The collected articles also emphasize that these effects are shaped by exposure timing, chemical mixtures, endocrine signaling, oxidative stress, metabolic remodeling, microbiome changes, and epigenetic regulation. Future studies should place greater emphasis on longitudinal human evidence, environmentally relevant mixtures, sex-specific responses, and experimental designs that can separate direct developmental toxicity from true transgenerational inheritance. Such work will be important for improving EDC risk assessment and for developing prevention strategies that protect both current and future generations.
